# Tocilizumab improves the efficacy of anti-PD-1 in a patient with advanced gastroesophageal junction cancer: a case report

**DOI:** 10.3389/fonc.2025.1530387

**Published:** 2025-04-04

**Authors:** Yushi Cai, Xuan Jin, Yun Dai

**Affiliations:** ^1^ Department of Gastroenterology, Peking University First Hospital, Beijing, China; ^2^ Department of Oncology, Peking University First Hospital, Beijing, China

**Keywords:** cancer-associated inflammation, immune checkpoint inhibitor, IL-6, tocilizumab, chemotherapy

## Abstract

**Background:**

Cancer-related inflammation contributes to the progression of malignancies and considerably affects therapeutic outcomes. IL-6 acts as a main mediator of both local and systemic inflammatory responses. Although IL-6 therapies have been successful in the treatment of inflammatory conditions, there has been little experience in patients with cancer.

**Case presentation:**

A 66-year-old man was diagnosed with gastroesophageal junction squamous cell carcinoma (stage IV) with liver metastasis. The patient presented with notable cancer-associated systemic inflammatory symptoms, and experienced disease progression after initial two cycles of anti-PD-1 combined with chemotherapy. After tocilizumab treatment, the symptoms improved rapidly. The patient showed favorable response to subsequent anti-PD-1 plus second-line chemotherapy, and survived without disease progression.

**Conclusion:**

Targeting IL-6 holds promise for the management of cancer-associated inflammation and improvement of therapeutic outcomes.

## Introduction

Although immune checkpoint inhibitors (ICIs) are established as effective treatments for various types of advanced cancer, overcoming therapeutic resistance remains a critical challenge. Accumulating evidence has established that systemic and local inflammatory status can considerably affect the antitumor immune responses and ICI efficacy (1). The inflammatory signals direct the tumor microenvironment and promote immunosuppression by recruiting suppressive cells and releasing soluble factors. Previous preclinical and clinical studies have shown that the inflammatory cytokines and chemokines have critical roles in tumor progression and are one of the major factors causing immunotherapy failure (2). Therefore, targeting inflammatory pathways can restore immune surveillance and may be a reasonable direction for cancer treatment. Interleukin 6 (IL-6) is a pleiotropic proinflammatory cytokine and acts as a main mediator of both local and systemic cancer-associated inflammatory responses (3). IL-6 expressed at high level in the tumor microenvironment promotes tumor cell proliferation, survival, invasion and metastasis. Importantly, IL-6 induces strong immunosuppression in the tumor microenvironment by recruiting immunosuppressive cells and impairing T cell infiltration (4). Recent study has identified IL-6 as a predictive factor of poor response to ICIs in large clinical trials of several types of cancer (5). In preclinical models, combined blockade of PD-L1 and IL-6 receptor leads to synergistic regression of tumors and substantially improves antitumor CD8^+^ T cell responses compared with anti-PD-L1 alone (6, 7). This evidence indicates that inhibiting IL-6 or its downstream signaling pathways could overcome ICI resistance. However, the potential therapeutic effects of blocking IL-6 combination with ICIs are unknown in cancer patients.

Here, we report a patient with advanced gastroesophageal junction carcinoma who failed to respond to initial chemotherapy and anti-PD-1 therapy, but experienced significant remission after administration of tocilizumab, an anti-IL-6 receptor antibody.

## Case presentation

**Figure 1:**
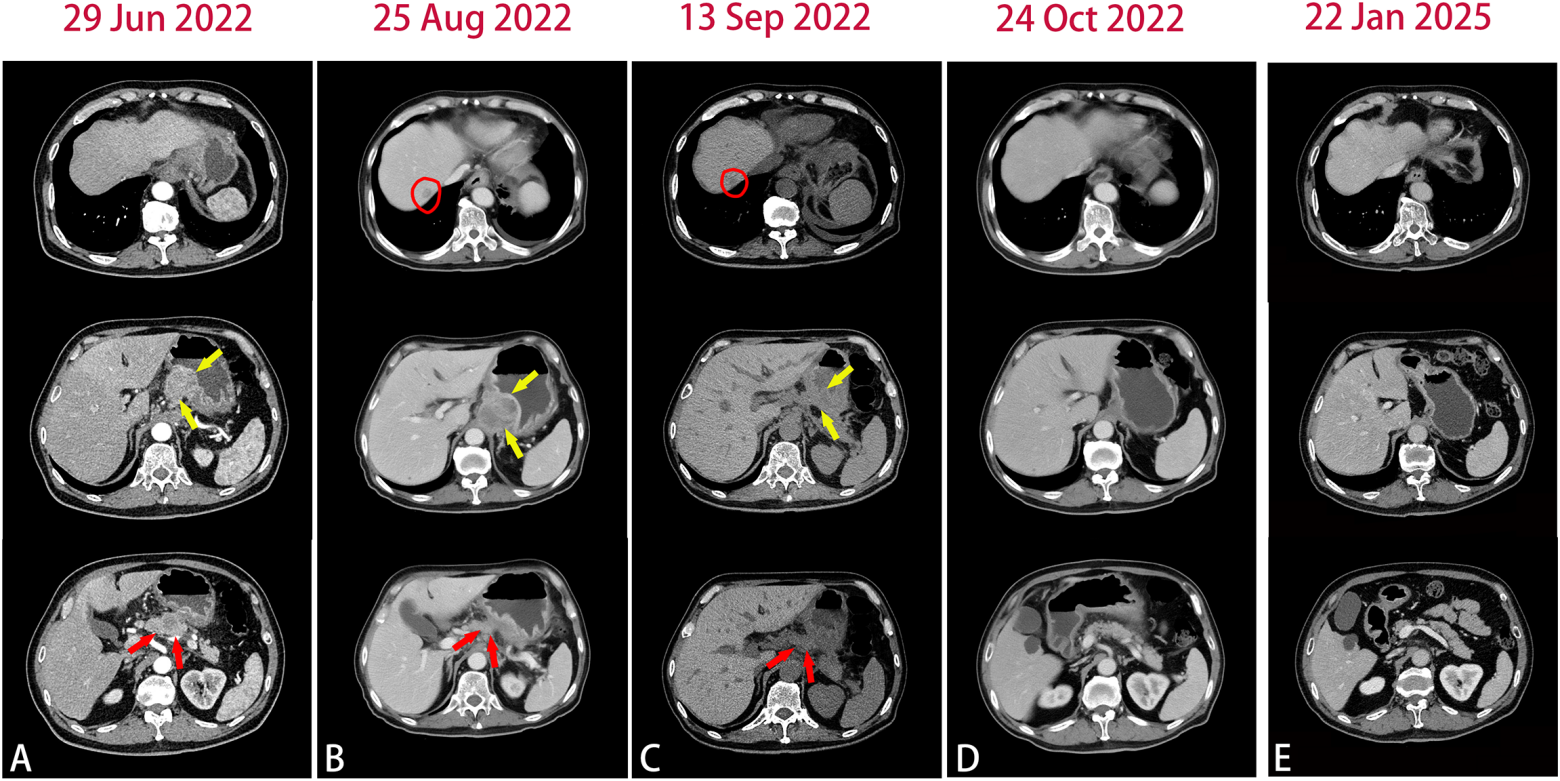
Abdomen CT images in the course of disease. **(A)** Baseline CT images showed the primary lesion (yellow arrow) and pancreas involvement (red arrow). **(B)** After 2 cycles of chemotherapy plus pembrolizumab, CT images showed newly emerged liver metastatic lesion(circle) and the primary lesion(yellow arrow) were slightly enlarged. **(C)** CT images after tocilizumab treatment. **(D)** After 2 cycles of second-line chemotherapy plus pembrolizumab, CT images showed significantly shrunk primary lesion and liver metastases. **(E)** The most recent CT images.

**Figure 2:**
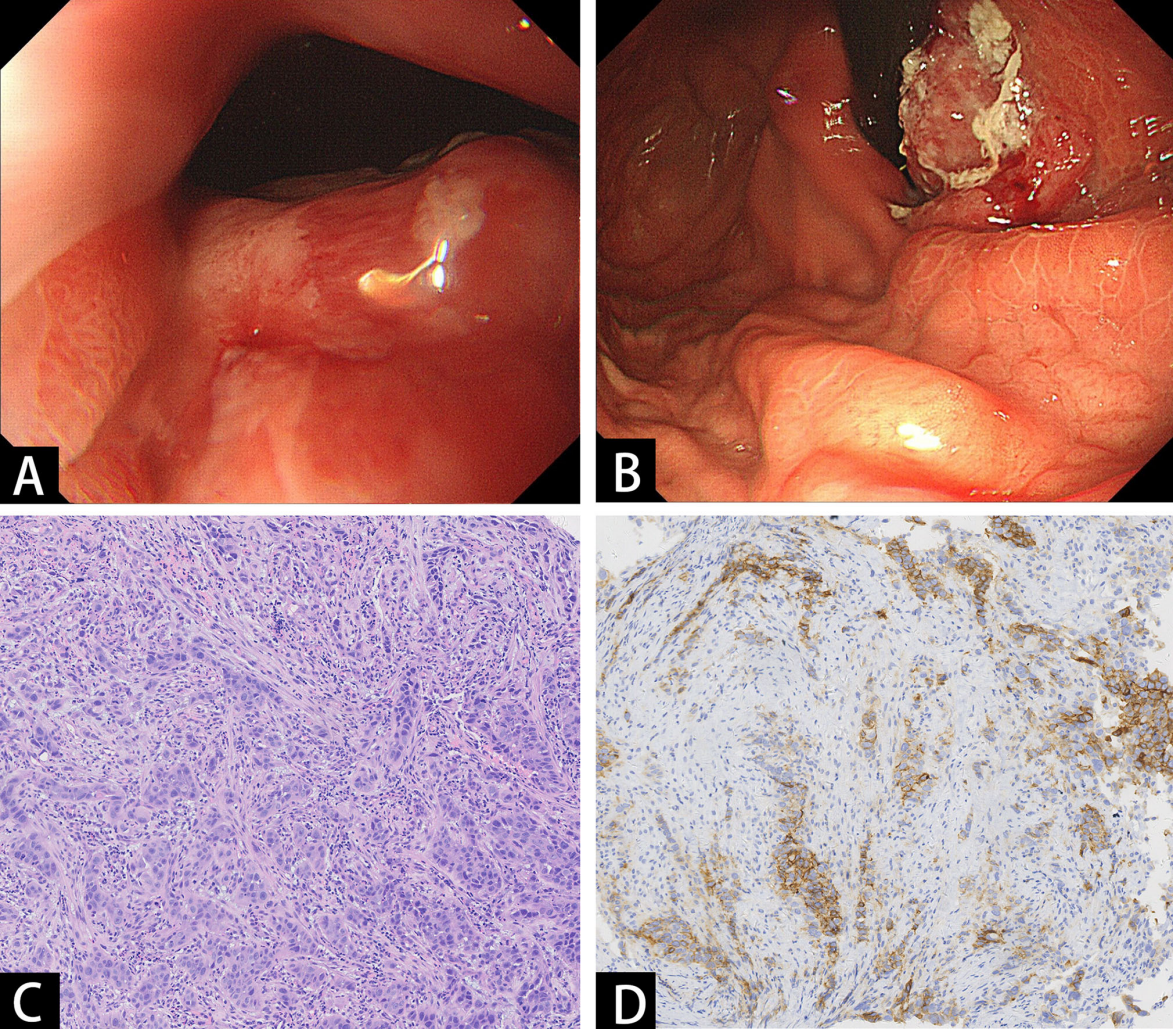
Endoscopic and pathologic findings of the tumor. **(A, B)** Endoscopy showed an ulcerative mass in the lower esophagus and cardia. **(C)** Histopathological examination showed poorly differentiated squamous carcinoma. **(D)** Immunohistochemistry staining of PD-L1.

**Figure 3:**
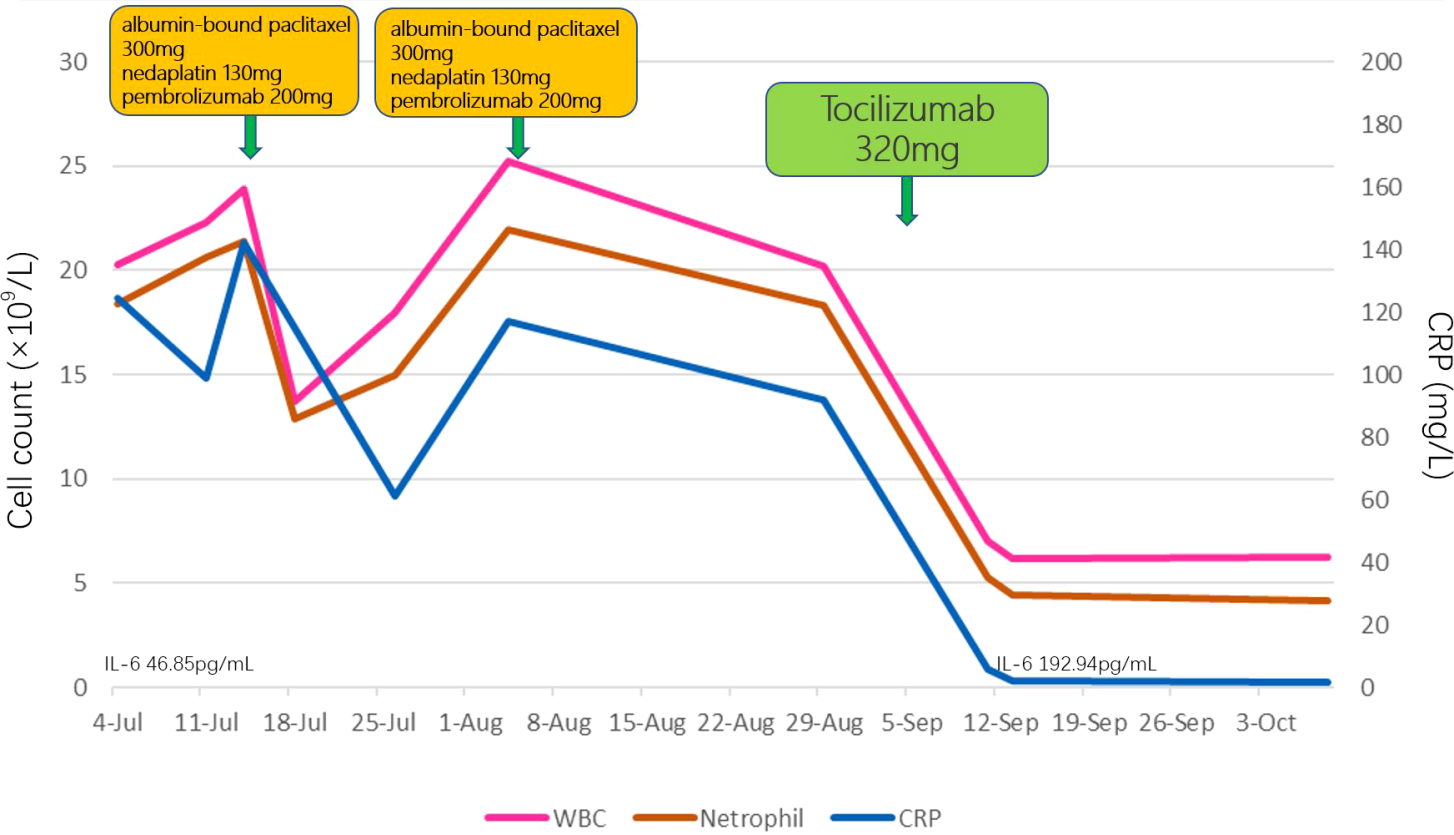
Levels of WBC, neutrophil, CRP and IL-6 and during the treatment.

**Figure 4:**
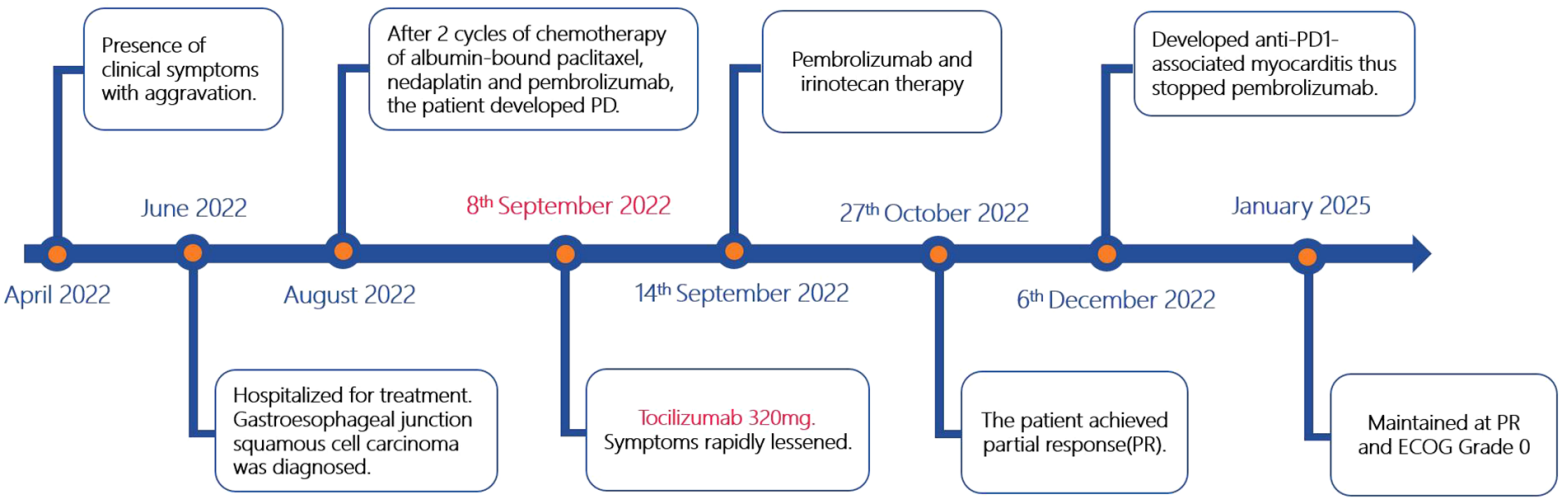
Timeline chart along with key dates for investigations and treatments of this patient.

A 66-year-old man with no significant past medical history presented to our hospital in June 2022 with a 2-month history of epigastric pain and intermittent melena. The patient also complained fatigue, night sweats, and a 5-kilogram loss of body weight. His Eastern Cooperative Oncology Group (ECOG) performance status was Grade 1. Physical examination revealed emaciation and tenderness in the mid-upper abdomen without rebound pain. Laboratory data showed an elevated white blood cell (WBC) count of 20.3×10^9^/L (neutrophil count of 18.4×10^9^/L and monocyte count of 0.9×10^9^/L), hemoglobin concentration of 101g/L, and platelet count of 404×10^9^/L. Serum chemistry showed total protein, albumin, and C-reactive protein (CRP) level of 66.2 g/L, 28.3 g/L and 124.62 mg/L, respectively. Serum IL-6 was also remarkably increased (46.85 pg/mL). The level of serum glycoantigen 125 (CA125) was slightly elevated (125 IU/ml), while other tumor indices including carcinoembryonic antigen (CEA), squamous cell carcinoma antigen (SCC) and cytokeratin 19 fragment (Cyfra21-1) were in the normal range. The immunoglobulin, complement and auto-antibodies were all normal. Enhanced computed tomography (CT) scan showed a soft tissue mass in the gastroesophageal junction with lymph nodes enlargement, and pancreas involvement (**Figure 1**). Endoscopy showed an ulcerative lesion in the lower esophagus and cardia, with lumen narrowing (**Figure 2**). Histopathological examination showed poorly differentiated squamous carcinoma. Immunohistochemistry results showed Ki67 (++, 50%), HER2 (-), EBV (-), pMMR (MLH1+, MSH2+, MSH6+, PMS2+) and PD-L1 CPS=40 (**Figure 2**). Based on these results, a diagnosis of gastroesophageal junction squamous cell carcinoma (T4bN2M1) was established. After the exclusion of infection by blood culture and testing for tuberculosis, viral and fungal infections, he received systemic chemotherapy (albumin-bound paclitaxel 300mg + nedaplatin 130mg) and pembrolizumab (200mg) on 15th July. However, after 2 cycles of therapy, the patient developed progressive disease (PD), with increased tumor volume and a newly emerged metastatic lesion in liver (**Figure 1**). In addition, the patient developed a fever of 38.5°C, and the symptoms deteriorated, with dysphagia, fatigue and night sweats. His ECOG performance status became Grade 3. Laboratory data also showed high levels of WBC count (25.21×10^9^/L) and CRP (116.83 mg/L). These clinical findings suggested a cancer-associated systemic inflammatory status, which may affect therapeutic outcomes. After agreement by the patient and his family and receipt of a written informed consent, we treated him with a single dose of tocilizumab (6.4mg/kg, 320 mg) on 8^th^ September. His temperature returned to normal within the first day of administration and the symptoms rapidly improved. The levels of WBC count and CRP decreased to normal range within 3 days, whereas IL-6 increased to 192.94 pg/mL (**Figure 3**). His overall physical condition recovered and ECOG performance status was improved to Grade 1 so that he received pembrolizumab (200 mg) and irinotecan (200 mg) therapy on 14^th^ September. Unexpectedly, the patient achieved partial response (PR) after 2 cycles of treatment, with significantly shrunk primary tumor and metastatic lesions (**Figure 1**). The level of CA125 also decreased to normal after treatment. Unfortunately, he developed anti-PD-1-associated myocarditis on 6^th^ December 2022 and the treatment was discontinued. After recovery, the patient declined surgery and systemic chemotherapy, but opted for oral anlotinib antiangiogenic therapy. During the follow-up period, he continued anlotinib monotherapy without other treatment including herbal medicine. His disease evaluation was maintained at PR and ECOG performance status was Grade 0 till now (more than 28 months after anti-PD-1 and second-line chemotherapy). Treatment and follow-up are organized into a timeline (**Figure 4**).

## Discussion

Inflammation is a hallmark of cancer that substantially contributes to the development and progression of malignancies. However, the effects of cancer-related inflammation extend beyond the tumor site, with the mechanisms for the systemic effect remaining largely unknown. There are complex interactions between the local and systemic inflammation and tumor progression, which involve components of the adaptive and innate immune systems. Such interactions considerably alter the disease course of a wide range of solid tumors. Local inflammation, also considered as local immune response, plays multiple roles within the tumor microenvironment (1, 8). Tumor-infiltrating T cell-mediated cytotoxicity controls tumor growth, whereas tumor-derived and host-derived cytokines and inflammatory mediators could facilitate tumor cell proliferation, angiogenesis, metastasis and induce immunosuppression in tumor microenvironment. Systemic inflammation involves cytokines, small inflammatory proteins, circulating immune cells, contributing to the paraneoplastic symptoms and patient outcomes. Cancer-related inflammation is influenced by the tumor stage and clinical circumstance. Tumor-derived cytokines and growth factors are secreted into the systemic circulation to mediate communication with distant sites, inducing myelopoiesis as well the production of acute-phase proteins, coagulation, and complement factors in the liver. Cancer-related inflammation lacks a clear definition or specific biomarkers. The symptoms observed in patients mainly include fever, night sweats, fatigue, and body weight loss (1). The most extreme result of systemic inflammation is cancer cachexia, which frequently affects patients with advanced cancer and creates a big challenge to treatment. IL-6 as well as the plasma acute-phase proteins such as CRP, amyloid A, α1 antitrypsin and α1 acid glycoprotein are accepted markers of systemic inflammation (9). Previous studies that have shown an association between the survival and systemic inflammatory responses in solid tumors including gastric cancer, gastroesophageal cancer, and colorectal cancer (10–12). Thus, targeting cancer-related inflammation has the potential to alter the disease course and benefit patients (13).

Accumulating evidence has established that IL-6 plays a pivotal role in cancer-associated inflammation, and overexpression of IL-6 in the tumor site and increased circulating level of IL-6 are related with poor clinical prognosis in multiple types of cancer (14–16). The main sources of IL-6 in the tumor microenvironment are tumor cells, stromal cells and infiltrating immune cells. IL-6 activates JAK/STAT3 signaling pathway to promote inflammatory responses and support tumor progression (17). Chemotherapy and radiotherapy can increase the expression of IL-6 in tumors, resulting in resistance to the treatment (18, 19). Therapeutic strategy of IL-6 signaling blockade has been investigated in phase 1 and 2 clinical trials, such as siltuximab in ovarian and renal cancer, with some promising results (20, 21). Siltuximab can inhibit cytokine production, tumor angiogenesis and intratumoral macrophage infiltration in ovarian cancer. In addition, a case report has indicated the successful treatment of cancer cachexia in a patient with lung cancer by using tocilizumab (22). After 2 weeks of tocilizumab treatment, there was observable reduction in plasm CRP, and an improvement in body weight and performance status. However, there were no clinical studies reported combination of tocilizumab with ICIs in solid tumors.

In this case, the patient presented with notable cancer-associated systemic inflammatory symptoms at baseline, including fatigue, fever, night sweats and weight loss. The blood test also showed significantly increased WBC count, CRP and IL-6. Based on the high expression level of PD-L1, the patient initially received a regimen combining pembrolizumab with chemotherapy. However, this treatment was discontinued due to disease progression. The symptoms and inflammatory markers had no significant improvement, and the tumor continued to grow and metastasize. Cancer-related inflammation with related metabolic and nutritional changes contribute to immunosuppression and can thus considerably affect the antitumor immune responses and ICI efficacy. IL-6 signaling pathway plays a crucial role in maintaining a precancerous inflammatory status and potentially influences anti-PD-L1 efficacy through several mechanisms, including inhibition of CD8^+^ T cell cytotoxic differentiation (5), limitation of Th1 responses (23), promotion of immunosuppressive myeloid cells (24), and disruption of conventional dendritic cells (25). Recent study has identified high plasma IL-6 level as a feature of anti-PD-L1 resistance in patients with advanced cancers (26). Preclinical studies have also shown that IL-6R blockade or genetic ablation of CD8^+^ T cell intrinsic IL-6 signaling synergized with anti-PD-L1 therapy to enhance antitumor responses, leading to improved tumor control (6). Tocilizumab recognizes both membrane-bound and soluble IL-6 receptors and specifically blocks the binding of IL-6. This drug has been approved for the treatment of multiple inflammatory conditions including rheumatoid arthritis, juvenile idiopathic arthritis, Castleman’s disease, and severe chimeric antigen receptor (CAR) T cell-induced cytokine release syndrome (27–29). In this patient, the dose of tocilizumab was determined by extrapolation from other inflammatory diseases (8mg/kg). Due to the patient’s poor performance status, the dose was reduced by 20% (6.4 mg/kg). After a single dose of tocilizumab treatment, the symptoms rapidly relieved and inflammatory markers (WBC count and CRP) decreased. Six days later, his performance status was improved, allowing the patient to resume antitumor therapy. An unexpected and remarkable improvement in the response of the patient to subsequent anti-PD-1 and chemotherapy was noted following tocilizumab treatment, suggesting blocking IL-6 signaling may facilitate overcoming anti-PD-1 resistance. Indeed, tocilizumab treatment marked a “turning point” in disease course, as the patient remained in remission even though anti-PD-1 therapy was discontinued and the patient only received maintenance antiangiogenic therapy. Previous studies have shown that the median overall survival (OS) for anti-PD-1 combined with chemotherapy as first-line and second-line treatment in advanced or metastatic gastroesophageal junction cancer were 11.6~17.2 months and 7.2~10.9 months, respectively; the median progression free survival (PFS) for the first-line and second-line treatment were 5.7~8.3 months and 1.5~2.7 months, respectively (30). In this case, the PFS period has exceeded 28 months after anti-PD-1 plus second-line chemotherapy. We look forward to a more durable clinical benefit for the patient.

Tocilizumab has a half-life of 11~13 days (31), therefore the suitable administration schedule for this drug to concurrent with ICIs and chemotherapy needs to be investigated. The patient’s serum IL-6 level was increased after tocilizumab administration, as it is in rheumatoid arthritis and Castleman’s disease. This may be due to the fact that IL-6R-mediated consumption of IL-6 is inhibited by the unavailability of tocilizumab-free IL-6R (32). In addition, the role of IL-6 and the complexity of the use of IL-6 blockade in cancer therapy should be noted. Numerous studies have shown that signaling via the IL−6/JAK/STAT3 pathway induces the expression of PD−1 and PD−L1 (33, 34). Inhibition of IL−6/JAK/STAT3 signaling induced PD−1 and PD−L1 downregulation might have one of two possible effects on the efficacy of ICIs. On the one hand, targeting IL−6/JAK/STAT3 signaling might improve the efficacy of ICIs as a result of direct inhibitory effects on tumor cells as well as effects on immune cells in the tumor microenvironment. On the other hand, downregulation of PD−1 and PD-L1 could attenuate the activity of ICIs by reducing the expression of the proteins targeted by these antibodies (13). However, the meaningful benefits observed in this case suggest that intervention with tocilizumab is worth testing in patients with cancer-associated inflammation. Given the extensive clinical experience with IL-6 inhibitors, combination of tocilizumab with ICIs and chemotherapy warrants investigation in future clinical studies.

## Data Availability

The original contributions presented in the study are included in the article/supplementary material. Further inquiries can be directed to the corresponding author.
